# miR-369-3p Modulates LRRK2-Mediated Inflammation and Autophagy in RAW264.7 Macrophages

**DOI:** 10.3390/ijms27073220

**Published:** 2026-04-02

**Authors:** Viviana Scalavino, Emanuele Piccinno, Ilaria Grassi, Raffaele Armentano, Gianluigi Giannelli, Grazia Serino

**Affiliations:** National Institute of Gastroenterology S. De Bellis, IRCCS Research Hospital, Via Turi 27, 70013 Castellana Grotte, Bari, Italy; viviana.scalavino@irccsdebellis.it (V.S.); emanuele.piccinno@irccsdebellis.it (E.P.); ilaria.grassi@irccsdebellis.it (I.G.); raffaele.armentano@irccsdebellis.it (R.A.); gianluigi.giannelli@irccsdebellis.it (G.G.)

**Keywords:** autophagy, immune response, ulcerative colitis, LRRK2, miR-369-3p

## Abstract

Leucine-rich-repeat kinase 2 (LRRK2) is a multidomain protein highly expressed in immune cells and implicated in the regulation of immune functions including immune signaling, cytokine release and autophagy. LRRK2 is one of the therapeutic targets in Parkinson’s Disease (PD). Aberrant activation of LRRK2 can also contribute to intestinal inflammation, mainly in inflammatory bowel disease (IBD). Hence the modulation of LRRK2 may influence gut inflammation providing an improvement in disease outcomes. Over the years, microRNAs (miRNAs) have acquired much attention thanks to their potential as therapeutic targets in several pathological conditions, including inflammatory disorders. In this study, we aimed to examine the ability of miR-369-3p in the modulation of autophagy targeting LRRK2 expression. Bioinformatics analysis revealed that Lrrk2 is a target gene of miR-369-3p, and LRRK2 expression was increased in ulcerative colitis patients compared with that in a healthy control. In in vitro analysis, we validated that mimic transfection with miR-369-3p in Raw264.7 significantly reduced the expression of LRRK2 both in basal and inflammatory conditions. Moreover, the inhibition of LRRK2 limited the nuclear translocation of Nuclear factor kappa B (NF-κB) induced by lipopolysaccharide (LPS) stimulation. In turn, we found that, in inflammatory conditions, the intracellular increase in miR-369-3p precluded the inhibition of autophagy by LRRK2 by restoring autophagy marker light chain 3 (LC3)II/I ratio, BECLIN-1 and decreasing p62 expression. Furthermore, we detected that the upregulation of miR-369-3p decreased the release of pro-inflammatory mediators Tumor necrosis factor (TNF), C-C motif ligand 2/Monocyte chemoattractant protein-1 (CCL2/MCP-1), C-C motif ligand 3/Macrophage inflammatory protein-1 alpha (CCL3/MIP-1α) and C-C motif ligand 5/Regulated on activation, normal T-cell expressed and secreted (CCL5/RANTES) and increased the anti-inflammatory cytokine interleukin 10 (IL-10) in response to LPS. This study supports the anti-inflammatory potential of miR-369-3p in immune cells and suggests the potential of miR-369-3p as a therapeutic agent in the treatment of acute intestinal inflammatory conditions such as ulcerative colitis.

## 1. Introduction

Leucine-rich-repeat kinase 2 (LRRK2) is a large multidomain protein that is highly expressed in various antigen-presenting cells and plays a crucial role in immune signaling [1]. Structurally, LRRK2 consists of a catalytic core, composed of a kinase domain, referred to as “KIN”, and a GTPase domain known as the Ras Of Complex proteins or ROC. These domains are connected via the C-terminal “COR” domain of ROC and are flanked by protein–protein interaction domains. The N-terminus comprises the armadillo, ankyrin, and leucine-rich-repeat (LRR) domains, while the C-terminus contains the WD40 domain, which is fundamental for protein folding [2]. LRRK2 has two main enzymatic functions within its catalytic core: GTPase activity and serine/threonine kinase activity [3,4]. LRRK2 is involved in several cellular processes, including vesicle trafficking, mitochondrial function, cytoskeleton dynamics, autophagy, phagocytosis and innate immune signaling [1,5,6].

Previous discoveries have identified LRRK2 as a key determinant in the pathophysiology of Parkinson’s Disease (PD). Specifically, various pathogenic mutations in LRRK2 at the catalytic core level are linked to PD pathogenesis, as they increase the kinase activity of LRRK2 [7]. In addition, the elevated expression of LRRK2 in immune cells has been directly associated with pro-inflammatory signals [8]. Notably, the aberrant expression of LRRK2 has also been connected to inflammatory diseases, including leprosy, tuberculosis and inflammatory bowel disease (IBD) [1,8,9], and cancer [10,11].

IBD, which includes Crohn’s disease (CD) and ulcerative colitis (UC), consists of chronic conditions characterized by persistent and recurrent inflammation affecting the gastrointestinal tract [12]. In healthy conditions, the innate immune system coordinates and modulates the host defense against triggers, ensuring a balance between tolerance and immunity [13]. In IBD, the aberrant immune response, in combination with genetic and environmental factors and altered gut microbiota, plays a crucial role in disease development and progression [12]. In the active phase of disease, the altered immune activity disrupts the balance between tolerance and immunity, triggering an inflammatory cascade that can affect both immune and non-immune cells, thereby causing chronic inflammation and damage to the intestinal mucosa [14,15].

Several studies have demonstrated the involvement of LRRK2 in IBD, although its role is still controversial. Lrrk2 has been shown to negatively regulate the Nuclear factor of activated T-cells 1 (NFAT-1) transcription factor, and its inhibition increased susceptibility to the development of dextran sulfate sodium (DSS)-induced colitis through increased activity of NFAT-1 [16]. Conversely, other studies have reported the association between increased LRRK2 expression and a more severe form of colitis. In particular, a study in preclinical models of colitis in mice demonstrated that the inhibition of LRRK2 contributed towards a reduction in the development and progression of colitis, promoting the polarization of macrophages toward a protective phenotype [17]. Another study demonstrated that LRRK2 inhibition ameliorated DSS-induced colitis by decreasing the production of Dectin-1-mediated pro-inflammatory cytokines and restoring autophagic processes [18]. Takagawa and colleagues examined the ability of several LRRK2 inhibitors to inhibit the production of inflammatory cytokines, identifying some LRRK2 inhibitors capable of reducing TNF production in both mouse bone marrow-derived dendritic cells (BMDCs) and human dendritic cells (DCs) derived from CD patients. Moreover, the application of LRRK2 inhibitors in murine models of colitis (LRRK2-IN-1 and GNE-7915) improved DSS-induced colitis [18]. Despite these studies supporting the involvement of LRRK2 in the pathogenesis of IBD, its role still needs to be fully elucidated.

Over the years, there has been a growing focus on post-transcriptional regulatory mechanisms that may influence inflammatory pathways involved in IBD, particularly those involving microRNAs (miRNAs). MiRNAs are 22–24-nucleotide small non-coding RNAs involved in the regulation of a broad range of biological processes by targeting different messenger RNAs (mRNAs) at the post-transcriptional level [19]. Several studies demonstrated that miRNAs modulate the immune cell functions, affecting many aspects of immune responses [20,21,22,23]. Notably, miRNAs play a key role in the pathogenesis of IBD. The impaired expression of miRNAs leads to the alteration of different molecular pathways that drive chronic intestinal inflammation [23,24]. Thus, the modulation of miRNAs’ expression may represent a strategy to ameliorate the inflammatory response that characterizes IBD.

LRRK2 has emerged as an important regulator of the autophagy pathway in immune cells [1]. Autophagy is a well-conserved mechanism of intracellular homeostasis involved in the body’s immune defense functions [25]. In the innate and adaptive immune systems, autophagy supports immune cell maintenance, function, and response. Specifically, autophagy contributes to the regulation of the immune response via pattern recognition receptor (PRR)- and NOD-like receptor (NLR)-mediated signaling pathways, which are essential in detecting pathogens and triggering inflammatory responses [26,27]. However, autophagy dysregulation is implicated in inflammatory diseases. In IBD, improper autophagy may play a role in the disease’s pathogenesis, affecting intestinal barrier maintenance, immune regulation, and microbial homeostasis [25,27]. Notably, several studies have identified miRNAs as crucial regulators of autophagy via various molecular pathways [25,28,29]. Therefore, the modulation of LRRK2 at post-transcriptional levels may influence the regulation of immune-mediated inflammatory responses and the inflammatory processes associated with IBD.

In this study, we assessed the role of miR-369-3p in the regulation of inflammatory response and autophagy via LRRK2 inhibition. We demonstrated that, in macrophages, an increase in miR-369-3p levels reduced the expression of LRRK2 in both basal and inflammatory conditions. In turn, a reduction in LRRK2 restricted the lipopolysaccharide (LPS)-induced nuclear translocation of Nuclear factor kappa B (NF-κB). Moreover, our findings revealed that the inhibition of LRRK2 by miR-369-3p influenced the autophagy pathway, restoring the autophagy marker light chain 3 (LC3)II/I ratio and BECLIN-1 expression and decreasing the p62 expression impaired by inflammatory conditions. Lastly, the upregulation of miR-369-3p decreased the release of pro-inflammatory mediators and increased anti-inflammatory effector release. Collectively, our findings confirm the anti-inflammatory effects of miR-369-3p in immune cells, pointing out its potential contribution to improving acute intestinal inflammatory disorders, including UC.

## 2. Results

### 2.1. Lrrk2 as a Putative Target of miR-369-3p

**Figure 1:**
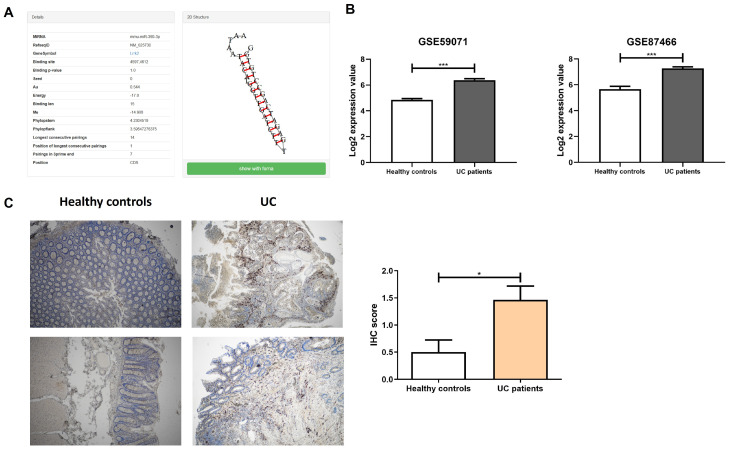
The Lrrk2 gene is a putative target of miR-369-3p overexpressed in UC patients. (**A**) Bioinformatic identification of miR-369-3p putative target gene Lrrk2 using the miRWalk algorithm. miR-369-3p interacts with Lrrk2 mRNA through the CDS region. (**B**) LRRK2 expression in colonic tissue from UC patients and healthy controls downloaded from the GEO databases (GSE59071 and GSE87466). Mean expression data are show as log of expression values. (*** *p* < 0.001). (**C**) Representative images of immunohistochemical assays for LRRK2 expression in tissues obtained from UC patients and healthy controls. IHC score representing the expression levels of LRRK2 protein in the immune infiltrate (* *p* < 0.05). Original magnification, 4×. Scale bar, 100 μm.

In our recent papers, we investigated putative target genes of miR-369-3p within the 3′ untranslated (3′ UTR) region. In the present study, we have extended our analysis to also include the 5′ untranslated (5′ UTR) region and the coding sequence (CDS) region. Accordingly, we conducted a bioinformatics analysis using the miRWalk algorithm [30]. Our analysis revealed that miR-369-3p could modulate Lrrk2 expression by binding to the CDS region (Figure 1A).

Over the years, LRRK2 has been reported to be associated with increased susceptibility to IBD. Furthermore, the altered expression of LRRK2 affects disease pathophysiology by regulating the immune response in the presence of intestinal inflammation [17,31]. To investigate this further, we analyzed two different public datasets downloaded from the Gene Expression Omnibus database (GSE59071 and GSE87466) to determine the expression levels of LRRK2 in UC patients (Figure 1B) [32,33]. These datasets presented gene expression profiles from 97 UC patients and 11 healthy controls, as well as from 87 UC patients and 21 healthy controls. Our analysis revealed that LRRK2 expression was enhanced in the inflamed intestinal mucosa of UC patients when compared with the normal mucosa of healthy controls (Figure 1B; *p* < 0.001).

We subsequently examined the protein expression of LRRK2 in our cohort of active UC patients and healthy controls enrolled at our institute. Tissue specimens from UC patients exhibited an overexpression of LRRK2 in comparison to healthy controls. Furthermore, the protein expression was predominant in the immune infiltrate (Figure 1C). A histological score was used to determine the grade of immunostaining. We found that, in UC patients, LRRK2 expression was significantly higher than in healthy controls (*p* < 0.0001; Figure 1C).

### 2.2. miR-369-3p Modulates LRRK2 Expression

**Figure 2:**
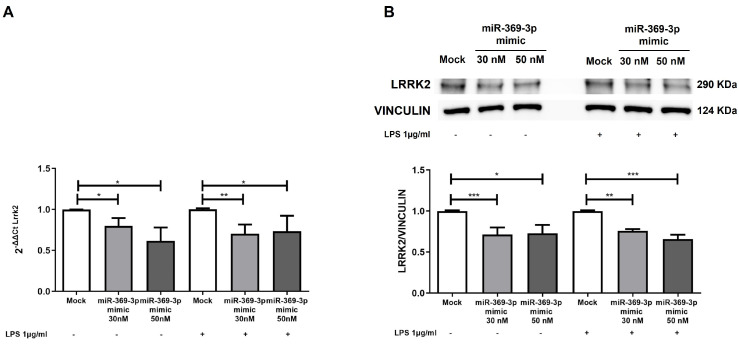
LRRK2 downregulation at mRNA and protein expression levels by miR-369-3p in Raw264.7 cells. (**A**) The mRNA expression levels of Lrrk2 were assessed by qRT-PCR in Raw264.7 cells transiently transfected with miR-369-3p mimic at concentrations of 30 nM and 50 nM. The Gapdh gene was used to normalize the Lrrk2 gene expression levels. (**B**) Western blot analysis performed to evaluate the expression levels of LRRK2 protein after transient transfection with miR-369-3p mimic in Raw264.7 cells at 30 nM and 50 nM miRNA concentrations, both basal and LPS-stimulated conditions. The protein levels were normalized to the housekeeping protein VINCULIN. Data are shown as the mean ± SEM representative of four independent experiments (* *p* < 0.05; ** *p* < 0.01; *** *p* < 0.001).

To assess whether miR-369-3p regulates the predicted target gene Lrrk2, identified through bioinformatic analysis, we conducted in vitro experiments using mouse Raw264.7 macrophages. Cells were subjected to transient transfection with the miR-369-3p mimic at concentrations of 30 nM and 50 nM, respectively, and then exposed to LPS stimulation for 6 h. The gene expression of Lrrk2 detected with real-time PCR (RT-PCR) revealed that an increase in intracellular miR-369-3p reduced Lrrk2 expression at both miRNA mimic concentrations (Figure 2A; *p* < 0.05). Similarly, following the LPS-induced inflammatory condition, the transient transfection with miR-369-3p significantly decreased the Lrrk2 gene expression (Figure 2A; *p* < 0.05). Subsequently, we performed Western blot analysis to evaluate the protein expression of LRRK2 after mimic transfection with miR-369-3p. As shown in Figure 2B, under basal conditions and following LPS stimulation, elevated levels of miR-369-3p modulated the protein expression of LRRK2 at both 30 nM and 50 nM mimic concentrations in comparison to the mock control (Figure 2B; *p* < 0.05).

### 2.3. Effect of miR-369-3p on NF-κB Activation and Translocation Through LRRK2 Regulation

**Figure 3:**
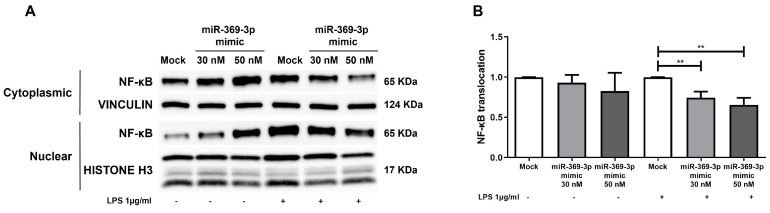
LRRK2 modulation by miR-369-3p impacted the activation and nuclear translocation of NF-κB upon LPS stimulation in Raw264.7. (**A**) Immunoblotting of cytoplasmic and nuclear fractions for NF-κB. (**B**) The histogram displays the ratio of nuclear NF-κB p65 over cytoplasmic NF-κB p65 content, which corresponds to the translocation of NF-κB from the cytoplasmic to nuclear compartment after miR-369-3p transfection and LPS stimulation. Protein levels were normalized using the housekeeping proteins VINCULIN and all three identified bands of HISTONE H3. Data are representative of four independent experiments. The histograms correspond to the mean ± SEM (** *p* < 0.01).

Prolonged and out-of-control NF-κB activation contributes to the pathogenesis of IBD [34]. Since LRRK2 has been implicated in NF-κB signaling as a positive regulator, promoting the release of pro-inflammatory cytokines [18], we examined the NF-κB pathway activation by analyzing the nuclear translocation of NF-κB in Raw264.7. Under LPS-mediated inflammatory conditions, the restricted expression of LRRK2 related to the intracellular enhancement of miR-369-3p significantly reduced the translocation of NF-κB from the cytosol to the nucleus at both 30 nM and 50 nM miRNA mimic concentrations compared with the mock control (Figure 3B; *p* < 0.01).

### 2.4. The Effects of LRRK2 Modulation on Autophagy

Autophagy plays a crucial role in the regulation of immune response. In IBD, the induction of autophagy mitigates the onset and progression of the disease, contributing to a reduction in inflammation and the maintenance of intestinal mucosal homeostasis [35]. Previous studies have suggested that LRRK2 is involved in the regulation of the autophagy pathway. An increase in LRRK2 expression suppressed autophagy and enhanced LRRK2-mediated inflammation [1,6,18].

**Figure 4:**
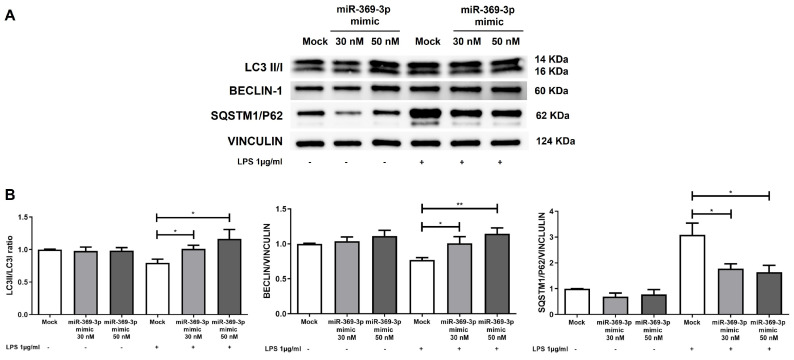
Transient transfection with miR-369-3p mimic improved the autophagic pathway. (**A**) Representative blots of LC3II/I, BECLIN-1, and SQSTM1/p62 protein expressions after miR-369-3p mimic transfection in Raw264.7 cells without and with LPS stimulation. (**B**) The histograms reported the quantitative analysis of LC3II/I ratio, BECLIN-1 and SQSTM1/p62 after miR-369-3p mimic in unstimulated and LPS-stimulated cells. Protein levels were normalized to the housekeeping protein VINCULIN. Data are representative of four independent experiments. The histograms correspond to the mean ± SEM (* *p* < 0.05; ** *p* < 0.01).

To determine whether the modulation of LRRK2 following miR-369-3p mimic transfection acted on the restoration of autophagy, we assessed the protein expression of components involved in the autophagy pathway. Our findings revealed that, compared with basal conditions, LPS stimulation reduced the ratio of LC3-II/LC3-I and BECLIN-1 expression associated with overexpression of p62. However, the intracellular elevation of miR-369-3p significantly improved the LC3-II/LC3-I ratio and BECLIN-1 expression, while p62 expression was considerably reduced at 30 nM and 50 nM mimic concentrations in comparison to the mock control (Figure 4B; *p* < 0.05).

### 2.5. miR-369-3p Promoted the Restoration of Inflammatory Effectors by Controlling LRRK2 Expression

**Figure 5:**
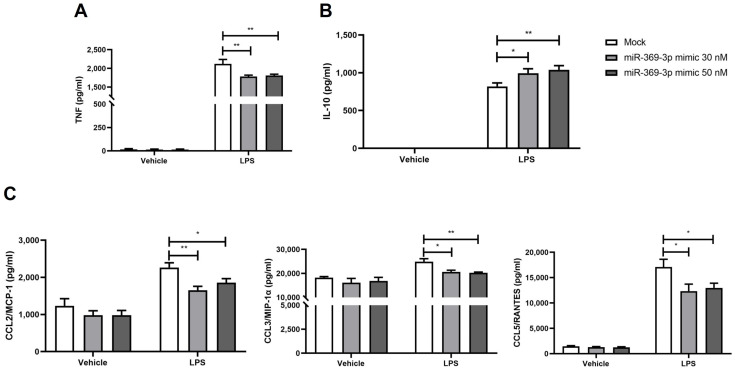
Pro-inflammatory and anti-inflammatory cytokines modulated by an intracellular increase in miR-369-3p mimic. Transient transfection of Raw264.7 with miR-369-3p mimic resulted in a significant decrease in pro-inflammatory cytokine TNF (**A**), a significant increase in anti-inflammatory cytokine IL-10 (**B**) and significant decrease in chemokines CCL2/MCP-1, CCL3/MIP-1α and CCL5/RANTES (**C**) in response to LPS stimulation. Data are representative of four independent experiments (* *p* < 0.05; ** *p* < 0.01).

In the presence of LRRK2 hyperactivity, immune cells released pro-inflammatory mediators, leading to an increase [36]. Based on this evidence, we investigated whether the modulation of LRRK2 by miR-369-3p regulated the secretion of inflammatory cytokines in Raw264.7 cells. After miR-369-3p mimic transfection and LPS stimulation, we observed an increased expression of TNF in the mock control. Despite this, the elevation of miR-369-3p significantly reduced the release of TNF at both 30 nM and 50 nM mimic concentrations (Figure 5A; *p* < 0.05). In contrast, the anti-inflammatory cytokine interleukin 10 (IL-10) significantly increased following mimic transfection with miR-369-3p when compared with the mock control (Figure 5B; *p* < 0.05). Additionally, we evaluated the production of C-C motif ligand 2/Monocyte chemoattractant protein-1 (CCL2/MCP-1), C-C motif ligand 3/Macrophage inflammatory protein-1 alpha (CCL3/MIP-1α) and C-C motif ligand 5/Regulated on activation, normal T-cell expressed and secreted (CCL5/RANTES) chemokines. Also, in this case, following LPS stimulation, the cellular increase in miR-369-3p led to a statistically significant decrease in chemokine release compared with the mock condition at both mimic concentrations (Figure 5C; *p* < 0.05).

## 3. Discussion

In the gut, the action of innate immune cells is pivotal to maintaining tissue homeostasis since an opportune and effective immune response ensures the balance between tolerance and immunity. IBD compromises this balance, triggering a range of dysregulated signaling that culminates in chronic inflammation and damage to the colon tissue [14]. Therefore, dysregulated immune cells represent a promising therapeutic target in the treatment of IBD.

LRRK2 is an enzymatic protein primarily associated with the pathogenesis of PD, but it is also associated with immune-related disorders, including IBD [31]. LRRK2 expression was observed in several immune cells, specifically, its high expression was detected following pro-inflammatory stimuli [1]. This highlighted the involvement of LRRK2 as a regulator of several immune cell pathways, such as pro-inflammatory cytokine release, Mitogen-activated protein kinase (MAPK) and NF-κB signaling pathways, autophagy, and phagocytosis [1,36,37].

Previous evidence has revealed that LRRK2 can play a crucial role in the pathogenesis of IBD. Genetic variants of the LRRK2 gene were associated with an increased risk of susceptibility to IBD [38,39,40], highlighting how an altered action of LRRK2 may contribute to the development of chronic inflammation. Experimental studies have yielded controversial results on the involvement of LRRK2 in intestinal inflammation. A study reported that LRRK2 deficiency exacerbates inflammation in DSS-treated mice through increased NFAT1 activation in macrophages [16]. Another study revealed that the overexpression of LRRK2 induced more-severe colitis, and the application of LRRK2 inhibitors ameliorated DSS-induced colitis through the inhibition of Dectin-1 and the restoration of autophagy [18]. A further study reported an exacerbated LRRK2 expression associated with the development of more-severe colitis. The inhibition of LRRK2 expression mitigated disease progression, leading to an improvement in the intestinal bacterial structure, reduced inflammation, and promoted the polarization of macrophages toward an M2 phenotype [17]. Overall, it is evident that altered LRRK2 expression supports inflammation and immune cell dysfunction.

Over the years, different studies have suggested that miRNAs act as a potential regulator of IBD pathogenesis by regulating immune responses, restoring epithelial barrier integrity, and modulating key inflammatory signaling pathways [24]. To date, studies on LRRK2-associated miRNAs are mainly related to PD. In patients with sporadic PD, a reduction in miR-205 was associated with elevated levels of LRRK2 expression, while overexpression of this miRNA suppressed abnormal LRRK2 expression [41]. Similarly, miR-599 reduced LRRK2 expression and protected neurons from decreased viability [42]. Furthermore, a study has examined the miRNA expression profiles in plasma and cerebrospinal fluid from patients with sporadic PD, confirming a close correlation between microRNA alterations and LRRK2 expression levels [43].

Altered activation of LRRK2 in macrophages affects the autophagic process. Autophagy dysfunction contributes to the pathogenesis of IBD, since impaired autophagy leads to persistent immune response and chronic inflammation, resulting in intestinal epithelium damage [44,45]. Autophagy regulators were identified as potential molecules for the pharmacological treatment of IBD [45]. Additionally, several findings have demonstrated that microRNA-mediated post-transcriptional regulation of autophagic mechanisms exerts positive effects in the therapeutic management of IBD [29].

In this study, we aimed to demonstrate how the anti-inflammatory potential of miR-369-3p can regulate the expression of LRRK2 in macrophages modulating the inflammatory response and autophagy pathway. Our previous studies have already characterized the anti-inflammatory capability of miR-369-3p, which engages distinct signaling pathways. In immune cells, we have demonstrated that miR-369-3p can attenuate inflammation by modulating the expression of CCAAT/enhancer-binding protein beta (C/EBP-β) and Nitric oxide synthase 2 (NOS2) [46,47], as well as by impairing the assembly and activation of the immunoproteasome and NOD-, LRR-, and pyrin domain-containing protein 3 (NLRP3) inflammasome through the inhibition of Proteasome 20S Subunit beta 9 (PSMB9) and BRCA1/BRCA2-containing complex subunit 3 (BRCC3), respectively [48,49]. Likewise, miR-369-3p mitigated inflammatory conditions by downregulating the expression of Phosphodiesterase 4B (PDE4B). This resulted in the activation of the cyclic adenosine monophosphate (cAMP)–Protein kinase A (PKA)—cAMP response element-binding protein (CREB) axis [50]. We also extended our analysis to intestinal epithelial cells (iECs). An intracellular increase in miR-369-3p suppressed MAPK signaling through Mitogen-activated protein kinase kinase 1 (MEK1) inhibition, leading to reduced Extracellular signal-regulated kinase (ERK) pathway activation and apoptosis modulation in iECs in response to TNF [51].

We have demonstrated that mimic transfection with miR-369-3p reduced the mRNA expression of Lrrk2, which was identified as a putative miRNA target gene through bioinformatic analysis. The analysis of various public datasets revealed that LRRK2 was overexpressed in UC patients compared with healthy controls. Additionally, we confirmed that LRRK2 expression was higher in the colon tissues of UC patients relative to healthy controls, with a primary impact on immune infiltrate. We subsequently experimentally demonstrated that miR-369-3p mimic transfection efficiently reduced the mRNA and protein expression of LRRK2 in Raw264.7 both under basal conditions and upon LPS stimulation. LRRK2 can affect NF-κB signaling and the autophagy process [18]. Aberrant NF-κB activation is involved in the pathogenesis of IBD [34]. During an inflammatory response, the activated NF-κB mediates the recruitment of immune cells to safeguard against pathogens and tissues damage. However, the persistent activation of NF-κB results in the unregulated and prolonged production of inflammatory factors, thus leading to chronic inflammation [34,52]. Our results have proven that the inhibition of LRRK2 by increasing levels of endogenous miR-369-3p limits the nuclear translocation of NF-κB that is induced by LPS stimulation. Furthermore, autophagy is a crucial process for the maintenance of intestinal homeostasis by regulating the inflammatory response. However, dysregulation of this process results in impaired epithelial barrier integrity and an altered immune response that exacerbates intestinal inflammation [44,52]. An LPS-induced inflammatory response was associated with autophagy inhibition in macrophages. Specifically, a deficit in the autophagy pathway is associated with an excessive increase in p62 and a decreased ratio of LC3II/I [53]. This study has demonstrated that miR-369-3p mimic transfection restored the compromised autophagy process triggered by inflammatory stimulation, increasing the LC3II/I ratio and BECLIN-1 expression while decreasing p62 expression. Overall, these processes have contributed to suppressing the release of pro-inflammatory mediators, including TNF, CCL2/MCP-1, CCL3/MIP-1α and CCL5/RANTES, while promoting the release of the anti-inflammatory cytokine IL-10.

Over the years, several compounds have been identified that can inhibit LRRK2 and enhance the therapeutic response in PD, thereby driving the discovery of novel treatments. Several studies have evaluated the ability of different LRRK2 inhibitors to mitigate neuroinflammatory response in preclinical models of PD [8]. Four generations of LRRK2 inhibitors are under consideration for clinical trials; nevertheless, they present some limitations such as poor potency, poor penetration of the blood–brain barrier and poor half-life [54]. At present, four small-molecule LRRK2 inhibitors, DNL201, WXWH0226, NEU-723, and BIIB122, are in clinical trials for PD treatment. These compounds have demonstrated good tolerability with few adverse events in PD patients. Notably, BIIB122 has progressed to a phase III clinical trial [55]. Nevertheless, further validation is ongoing to fully confirm the therapeutic efficacy of these small molecules in the treatment of PD.

In the field of IBD, different studies have highlighted the therapeutic potential of LRRK2 inhibitors in in vivo models of DSS-induced colitis. Two DSS-induced colitis mice with two different inhibitors, in particular LRRK2-IN-1 and GNE-7915, significantly ameliorated inflammatory conditions, indicating their potential as therapeutic agents for IBD [18]. Additionally, Cabezudo and colleagues reported that the oral administration of MLi-2, a strong and selective LRRK2 inhibitor, relieved intestinal inflammation in DSS-induced colitis mice [40]. These studies suggest that selective LRRK2 inhibitors can be promising therapeutic candidates for IBD treatment. However, their efficacy has not yet been evaluated in clinical trials.

MiRNA-based therapies may represent a valid alternative to LRRK2 inhibitors. Rather than traditional drugs targeting a single protein, miRNAs are able to simultaneously regulate multiple genes, affecting entire disease-related gene networks. This capability offers a potentially highly specific approach for treating inflammatory conditions and other disorders, reducing adverse effects and pharmacological resistance.

This paper has limitations. This preliminary study conducted solely in an in vitro model reported the anti-inflammatory potential of miR-369-3p in macrophages by modulating LRRK2 expression and inflammatory signaling and restoring the autophagy process. To reinforce the translational potential of our findings, the direct interaction between miR-369-3p and LRRK2 needed to be confirmed via a reporter assay to fully rule out an indirect regulatory mechanism. Moreover, additional validations in human monocyte-derived macrophages, including THP-1 cells and primary human monocytes, would further support the conservation of the observed mechanisms across species. In addition, further investigations are needed to verify the anti-inflammatory ability of miR-369-3p via LRRK2 modulation in in vivo colitis models to clarify its therapeutic potential in a complex biological system.

In conclusion, this study highlights how miR-369-3p is capable of modulating the inflammatory response and restoring the autophagy pathway though LRRK2 modulation. This post-transcriptional regulation may represent a preliminary in vitro observation in the management of impaired inflammatory immune responses. While additional research is necessary to validate efficacy in more-complex model systems and to evaluate its prospective clinical application, the use of miR-369-3p in combination with existing therapies may enhance the management of inflammatory disorders, including IBD.

## 4. Materials and Methods

### 4.1. Cell Culture and Treatment

The Raw264.7 murine macrophage cell line, obtained from American Type Culture Collection (ATCC, Manassas, VA, USA), was maintained at 37 °C in a humidified atmosphere with 5% CO_2_ and cultured in Dulbecco’s modified Eagle medium (DMEM, Thermo Fisher Scientific, Waltham, MA, USA) supplemented with 10% heat-inactivated fetal bovine serum (FBS, Thermo Fisher Scientific, Waltham, MA, USA), 1% 10,000 µg/mL streptomycin and 10,000 U/mL penicillin (Thermo Fisher Scientific, Waltham, MA, USA), 1% 1M HEPES (Sigma-Aldrich, St. Louis, MO, USA), and 1% 100 mM sodium pyruvate (Sigma-Aldrich, St. Louis, MO, USA).

For treatment, cells (passages from 5 to 16) were seeded into 12-well plates at a density of 1 × 10^6^ cells/well. The following day, cells were subjected to transient transfection with miR-369-3p mimic (Life Technologies, Hilden, Germany) at a concentration of 30 and 50 nM using the TransIT-TKO Transfection Reagent (Mirus Bio LLC, Madison, WI, USA) according to the manufacturer’s instructions. After that, cells were stimulated with 1 μg/mL of lipopolysaccharide (LPS, Sigma-Aldrich, St. Louis, MO, USA) for 6 h and then lysed for RNA isolation and protein extraction. The mock control, representing the control in each transfection experiment, was composed with the same transfection reagent but an equal volume of PBS to substitute miRNA mimic.

### 4.2. RNA Extraction and Real-Time PCR

Total RNA was extracted from RAW 264.7 cells using TRIZol reagent (Invitrogen, Carlsbad, CA, USA) according to the manufacturer’s protocol. The RNA concentration was determined using the NanoDrop spectrophotometer (Thermo Fisher Scientific, Waltham, MA, USA) and 1 μg of total RNA was reverse-transcribed using the iScript Reverse Transcription Supermix (BioRad Laboratories, Hercules, CA, USA) based on the manufacturer’s instructions. The obtained cDNA was used to perform qPCR amplification reactions on a CFX96 System (Biorad Laboratories, Hercules, CA, USA) using SsoAdvanced Universal SYBR Green Supermix (BioRad Laboratories, Hercules, CA, USA) and the primers for Lrrk2 and Gapdh (Qiagen, Hilden, Germany) and, then, analyzed using Bio-Rad CFX Manager Software (version 3.1). The housekeeping gene Gapdh was used to normalize the Lrrk2 gene expression. Comparative real-time PCR analysis was performed using four independent replicates.

### 4.3. Immunoblot Analysis

Total proteins were isolated using T-PER Protein Extraction Reagent (Thermo Fisher Scientific, Waltham, MA, USA) enriched with the protease inhibitor cocktail (Sigma-Aldrich, St. Louis, MO, USA), whereas cytoplasmic and nuclear separations were obtained using NE-PER lysis buffers (Thermo Fisher Scientific, Waltham, MA, USA) according to the manufacturer’s instructions. The protein concentration was determined using Bradford’s protein assay (Biorad Laboratories, Hercules, CA, USA). Samples were separated using 7.5% and 4–20% Mini-PROTEAN TGX Stain-Free Protein Gels (Biorad Laboratories, Hercules, CA, USA) and then transferred onto PVDF membranes (Biorad Laboratories, Hercules, CA, USA).

For immunodetection, membranes were exposed to antibodies using iBind Automated Western Systems (Thermo Fisher Scientific, Waltham, MA, USA) according to the manufacturer’s protocol. The primary antibodies LRRK2 (#13046, Cell Signaling, Technology, Danvers, MA, USA, dilution 1:1000), LC3A/B (#12741, Cell Signaling, Technology, Danvers, MA, USA, dilution 1:1000), Beclin-1 (#3495, Cell Signaling, Technology, Danvers, MA, USA, dilution 1:1000), SQSTM1/p62 (ab109012, Abcam, Cambridge, UK, dilution 1:2500), NFκB p65 (#8242, Cell Signaling, Technology, Danvers, MA, USA, dilution 1:2000), Vinculin (#13901, Cell Signaling, Technology, Danvers, MA, USA, dilution 1:5000), Histone H3 (#4499, Cell Signaling, Technology, Danvers, MA, USA, dilution 1:1000), and secondary antibody goat anti-rabbit IgG-(H+L)-HRP conjugate (31466, Invitrogen, Carlsbad, CA, USA, dilution 1:2500) were used. Membranes were imaged using the ChemiDoc Imaging System (Biorad Laboratories, Hercules, CA, USA) and analyzed and quantified with Image Lab Software version 6.1.0 (Biorad Laboratories, Hercules, CA, USA). Vinculin and Histone H3 were used as a housekeeping control to normalize the protein expression. Protein expression analyses were performed using four independent experiments.

### 4.4. Inflammatory Cytokine and Chemokine Assays

The supernatants were collected after transfection and LPS stimulation for cytokine and chemokine analysis. The expression levels of TNF, IL-10, CCL2/MCP-1, CCL3/MIP-1α and CCL5/RANTES were quantified using ELISA kits (R&D Systems, Minneapolis, MN, USA) in accordance with the manufacturer’s instructions. The plates were read at an absorbance of 450 nm using the Multiskan SkyHigh Photometer (Thermo Fisher Scientific, Waltham, MA, USA).

### 4.5. Immunohistochemistry in UC Patients’ Samples

Intestinal specimens obtained during surgical procedures and embedded in paraffin were collected from patients with active UC (*n* = 20) with a mean age of 54 years (range 18–80 years; 74% male) and healthy controls (*n* = 10). UC samples include total or subtotal proctocolectomies from patients with longstanding ulcerative colitis refractory to medical therapies. Age- and sex-matched controls displayed no evidence of intestinal inflammation. Sections were stained with hematoxylin and eosin and examined by a pathologist to determine the samples’ adequacy and their morphologic and/or pathological features. For IHC procedures, 4 µm sections were cut and mounted on Apex Bond IHC slides (Leica Biosystems, Buffalo Grove, IL, USA), and IHC staining was conducted using the BOND III automated immunostainer (Leica Biosystems, Buffalo Grove, IL, USA). Tissue sections were incubated with anti-LRRK2 primary antibody (ab133518 Abcam, Cambridge, UK, dilution 1:200). The Bond Polymer Refine Detection Kit (Leica Biosystems, Buffalo Grove, IL, USA) was used as a chromogen reagent according to the manufacturer’s instructions. Samples were negative when the number of stained cells was less than 5%.

The expression of LRRK2 was determined through a score calculated as follows: 0, absent; 1, low expression; 2, moderate expression; 3, intense expression.

### 4.6. Bioinformatics and Statistical Analysis

Putative gene targets of miR-369-3p were predicted using the miRWalk algorithm (http://mirwalk.umm.uni-heidelberg.de/, accessed on 20 February 2025) [30].

Data were assessed using GraphPad prism software version 10.0.4. Statistical significance, according to normal distribution of data, was determined with two-tailed Student’s *t* test or Mann–Whitney, and data were indicated as mean ± SEM. Data were representative of four independent experiments. A value of *p* < 0.05 was considered statistically significant.

## 5. Patents

An Italian patent entitled “Pharmaceutical composition based on miR-369-3p as active ingredient for the treatment of chronic inflammatory disorders” (patent No 102018000007954) was issued on 3 August 2020 to the Ente Ospedaliero Specializzato in Gastroenterologia “Saverio de Bellis”.

## Data Availability

The raw data of all Western blot experiments presented in the study are openly available on FigShare at doi 10.6084/m9.figshare.31314568.

## Abbreviations

The following abbreviations are used in this manuscript:

LRRK2	leucine-rich-repeat kinase 2
PD	Parkinson’s disease
IBD	inflammatory bowel disease
miRNAs	microRNAs
NF-κB	nuclear factor kappa B
LPS	lipopolysaccharide
LC3 II/I	autophagy marker light chain 3 II/I
TNF	tumor necrosis factor
CCL2	(C-C motif) ligand 2
MCP-1	monocyte chemoattractant protein-1
CCL3	(C-C motif) ligand 3
MIP-1α	macrophage inflammatory protein-1 alpha
CCL5	chemokine (C-C motif) ligand 5
RANTES	regulated on activation, normal T-cell expressed and secreted
IL-10	interleukin 10
ROC	Ras of complex protein
COR	C-terminal of Roc
LRR	leucine-rich-repeat
CD	Crohn’s disease
UC	ulcerative colitis
NFAT-1	nuclear factor of activated T-cells 1
DSS	dextran sulfate sodium
BMDCs	bone marrow-derived dendritic cells
DCs	dendritic cells
mRNAs	messenger RNAs
PRRs	pattern recognition receptors
NLRs	NOD-like receptors
3’ UTR	3’ untranslated region
5’ UTR	5’ untranslated region
CDS	coding sequence region
RT-PCR	real-time PCR
MAPK	mitogen-activated protein kinase
C/EBP-β	CCAAT/enhancer-binding protein beta
NOS2	nitric oxide synthase 2
NLRP3	NOD-, LRR- and pyrin domain-containing protein 3
PSMB9	proteasome 20S subunit beta 9
BRCC3	BRCA1/BRCA2-containing complex subunit 3
PDE4B	phosphodiesterase 4B
cAMP	cyclic adenosine monophosphate
PKA	protein kinase A
CREB	cAMP response element-binding protein
iECs	intestinal epithelial cells
MEK1	mitogen-activated protein kinase kinase 1
ERK	extracellular signal-regulated kinase
